# Antifungal Activities of Volatile Secondary Metabolites of Four *Diaporthe* Strains Isolated from *Catharanthus roseus*

**DOI:** 10.3390/jof4020065

**Published:** 2018-05-30

**Authors:** Dong-Hui Yan, Xiaoyu Song, Hongchang Li, Tushou Luo, Guiming Dou, Gary Strobel

**Affiliations:** 1 Research Institute of Forest Ecology, Environment and Protection, Chinese Academy of Forestry, Hai Dian District, Beijing 100091, China; songxiaoyucaf@gmail.com (X.S.); lhc1994224@163.com (H.L.); dgmgogogo@126.com (G.D.); 2 Research Institute of Tropical Forestry, Chinese Academy of Forestry, Tianhe District, Guangzhou 510520, China; luots@126.com; 3 Department of Plant Sciences, Montana State University, Bozeman, MT 59717, USA

**Keywords:** endophytic fungi, *Diaporthe* spp., *Catharanthus roseus*, volatile organic compounds (VOCs), antifungal bioactivity, inhibition, terpene, pathogens

## Abstract

Four endophytic fungi were isolated from the medicinal plant, *Catharanthus roseus*, and were identified as *Diaporthe* spp. with partial translation elongation factor 1-alpha (*TEF1*), beta-tubulin (*TUB*), histone H3 (*HIS*), calmodulin (*CAL*) genes, and rDNA internal transcribed spacer (ITS) region (*TEF1*-*TUB*-*HIS*--*CAL*-ITS) multigene phylogeny suggested for species delimitation in the *Diaporthe* genus. Each fungus produces a unique mixture of volatile organic compounds (VOCs) with an abundant mixture of terpenoids analyzed by headspace solid-phase microextraction (SPME) fiber-GC/MS. These tentatively-detected terpenes included α-muurolene, β-phellandrene, γ-terpinene, and α-thujene, as well as other minor terpenoids, including caryophyllene, patchoulene, cedrene, 2-carene, and thujone. The volatile metabolites of each isolate showed antifungal properties against a wide range of plant pathogenic test fungi and oomycetes, including *Alternaria alternata*, *Botrytis cinerea*, *Colletotrichum gloeosporioides*, *Fusarium graminearum*, and *Phytophthora cinnamomi.* The growth inhibition of the pathogens varied between 10% and 60% within 72 h of exposure. To our knowledge, the endophytic *Diaporthe*-like strains are first reported from *Catharanthus roseus*. VOCs produced by each strain of the endophytic *Diaporthe* fungi were unique components with dominant monoterpenes comparing to known *Diaporthe* fungal VOCs. A discussion is presented on the inhibitive bioactivities of secondary metabolites among endophytic *Diaporthe* fungi and this medicinal plant.

## 1. Introduction

Many plants remain unexplored for their endophytic fungi and the potentially important products that they may produce [1]. *Catharanthus roseus* is known as a pharmaceutical plant containing rich anticancer alkaloids. The extracts of many organs of this plant also exhibit antimicrobial effects [2,3,4,5,6]. It turns out that *Catharanthus roseus* is host to a diverse group of endophytic fungi [7,8,9,10]. Some endopytic fungi were found to produce several metabolites biosynthesized by the host *Catharanthus roseus*. The endophytic fungi *Curvularia* sp. CATDLF5 and *Choanephora infundibulifera* CATDLF6 isolated from leaf issues were able to enhance leaf vindoline production content of *C. roseus* cv. *prabal* by 2.29–4.03 times through root inoculation [8]. Endophytic *Fusarium* spp. from stem issues seemed to facilitate the host plant to produce secondary metabolites [9]. Additionally, some endophytic fungi from the plant produced antimicrobial compounds. For example, the compounds hydroxyemodin, citreoisocoumarin, citreoisocoumarinol, and cladosporin from endophytic fungi of leaves were effective in inhibiting fungal pathogens [10]. *Diaporthe* are commonly found as endophytes in a wide range of plants around the world [11,12,13,14,15]. These endophytes are prolific producers of antimicrobial metabolites [15,16]. *D. endophytica* and *D. terebinthifolii*, isolated from the medicinal plants *Maytenus ilicifolia* and *Schinus terebinthifolius*, had an inhibitory effect against *Pseudomonas citricarpa* in vitro and in detached fruits [12,13]. The crude extracts of *Diaporthe* sp. MFLUCC16-0682 and *Diaporthe* sp. MFLUCC16-0693 exhibited notable antibacterial and antioxidant activities [14]. An endophytic *Phomopsis* (asexual state of *Diaporthe*) fungus isolated from the stems of *Ficus pumila*, exhibited broad-spectrum antimicrobial activity against Gram-positive and Gram-negative human and phytopathogenic bacteria and fungi [15]. Thus, the genus *Diaporthe* is a potential source of metabolites that can be used in a variety of applications [14]. However, endophytic *Diaporthe* fungi have not been recorded from *Catharanthus roseus* to the present.

Volatile organic compounds (VOCs) have noted biofumigative effects especially from the endophytic fungus—*Muscodor albus* [17]. These observations opened a unique venue for the application of endophytic microorganisms to the ecological-friendly biocontrol of pests [17]. The inhibitive bioactive compounds were also found in a few isolates of endophytic *Diaporthe* [18]. An endophytic *Phomopsis* isolate of *Odontoglossum* sp. in Northern Ecuador was reported to produce a unique mixture of volatile organic compounds (VOCs) with sabinene, 1-butanol, 3-methyl; benzeneethanol; 1-propanol, 2-methyl, and 2-propanone. The VOCs showed antifungal bioactivities on a wide range of plant pathogenic fungi, such as *Sclerotinia*, *Rhizoctonia*, *Fusarium*, *Botrytis*, *Verticillium*, *Colletotrichum* and oomycetes *Pythium*, and *Phytophthora* [18]. The PR4 strain of an endophytic *Phomopsis* obtained from the medicinal plant *Picrorhiza kurroa* also produced a unique set of bioactive VOCs inhibitive to plant pathogenic fungi growth. The dominant compounds in VOCs of the PR4 strain were reported as menthol, phenylethyl alcohol, isomenthol, β-phellandrene, β-bisabolene, limonene, 3-pentanone and 1-pentanol [19]. In view of the antimicrobial properties of the extracts from the medicinal plant *Cantharatus roseus*, and limited knowledge on endophytic *Diaporthe* species in this host, we conducted an investigation on the antifungal bioactivity of VOCs from four endophytic *Diaporthe* strains isolated from wild *Catharanthus roseus* in China. The combined sequences of five loci, elongation factor 1-alpha (*TEF1*), beta-tubulin (*TUB*), histone H3 (*HIS*), calmodulin (*CAL*) genes, and the rDNA internal transcribed spacer (ITS) region were used for the strains’ phylogenetic analyses within genus *Diaprothe*. Inhibitory bioactivity executed volatile organic compounds from the strains were observed on growths of tested plant pathogens in co-culture. Active components of VOCs were analyzed and inferred using headspace solid-phase microextraction (SPME) fiber-GC/MS and based on their reported properties.

## 2. Materials and Methods

### 2.1. Endophytic Fungal Isolation

The four endophytic fungi were isolated from wild plants, *Catharanthus roseus*, growing in the National Natural Conservation Area of TongGu Mountain, located in Wenchang city of Hainan Province. Several stem segments (5–10 cm in length) were collected for the eventual isolation of endophytes. Retrieving endophytic fungi followed a previously described procedure [20]. Briefly, the external tissues of segments were cleaned with tap water and scrubbed with 70% ethanol prior to excision of internal tissues. Then the segments were excised into smaller fragments about 0.2–0.5 cm in length. The fragments were thoroughly exposed to 75% ethanol for 60 s, 3% NaClO for 90 s, and sterile water for 60 s by agitation. The fragments at the last step were drained on sterile filter papers and put on water agar in Petri plates for growing endophytes. Further, pure isolates were obtained in potato dextrose agar media and stored on sterilized, inoculated barley seeds at 4 °C and −80 °C. The four fungi of interest were assigned with our laboratory acquisition number-ID FPYF3053-3056 and deposited in China Forestry Culture Collection Center assigned IDs of CFCC 52704-52707.

### 2.2. DNA Extraction, PCR, and Sequencing

Fungal genomic DNA was extracted from colonies growing on PDA for one week with the CTAB procedure [20]. The extracted DNA was further purified through Mini Purification kit (Tiangen Biotech (Beijing) Co., Ltd., Beijing, China) following the manufacture’s protocols. The DNA quality and concentration were determined with a NanoDrop 2000 (Thermo Fisher Scientific Inc., Waltham, MA, USA) after the DNA was checked with Genegreen nucleic acid dye (Tiangen Biotech (Beijing) Co., Ltd.) in an electrophoresis on 1% agarose gel stained under ultraviolet light. The extracted DNA was used as a template for the further PCR amplification ITS sequence and *TEF1*, *CAL*, *TUB*, and *HIS* genes regions. The primers were used to amplify the ITS targets, namely, the ITS1 and ITS4 [21], *TEF1* with EF1-688F/EF1-1251R [22], *CAL* with CL1F/CL2A or CAL563F/CL2A [23], *TUB* with T1/Bt-2b or Bt2a/Bt-2b [24,25], and *HIS* with HISdiaF/HISdiaR, sequences that were 5′-GGCTCCCCGYAAGCAGCTCGCCTCC-3 and 5′-ATYCCGACTGGATGGTCACACGCTTGG-3, respectively. All PCR reaction mixtures and conditions were followed as per the Taq PCR MasterMix kits (Tiangen Biotech (Beijing) Co., Ltd.) according to the manufacture’s protocol. A PCR reaction system consisted of 0.5 µL of each primer (10 µM), 3 µL (15–80 ng) of DNA template, 12.5 µL of 2 × Taq PCR MasterMix (Tiangen Biotech (Beijing) Co., Ltd.), and 8.5 µL of double distilled water in total of 25 µL. The ITS thermal cycling program was as follows: 94 °C for 5 min, followed by 35 amplification cycles of 94 °C for 60 s, 55 °C for 30 s and 72 °C for 1 min, and a final extension step of 72 °C for 5 min. The annealing temperature at 55 °C for 45 s was changed in this program for *CAL*, *β-tubulin* and *TEF* amplification. For amplification of *HIS*, the program was changed with a cycling program of 32 cycles and an annealing temperature at 55 °C for 60 s. PCR products were visualized on 1.5% agarose gels mixed with Genegreen Nucleic Acid Dye and purified with a quick Midi Purification kit (Tiangen Biotech (Beijing) Co., Ltd.) according to the manufacturer’s instructions. Sequencing PCR products were cycle-sequenced the BigDye^®^ Terminator Cycle Sequencing Kit v. 3.1 (Applied Biosystems, Foster City, CA, USA) in an ABI Prism 3730 DNA Sequencer (Applied Biosystems, Foster City, CA, USA) at Biomed Company in Beijing. Then sequence data collected by ABI 3730 Data collection v. 3.0 (Applied Biosystems, Foster City, CA, USA) and ABI Peak Scanner Software v. 1.0 (Applied Biosystems, Foster City, CA, USA), were assembled with forward and reverse sequences by BioEdit. The gene sequences were submitted and awarded access numbers in GenBank of NCBI (Table 1).

### 2.3. Sequence Alignment and Phylogenetic Analysis

In order to determine the phylogenetic locations of the four isolates within the *Diaporthe* genus, 143 reference taxa [26] (Table 2) together with the four isolates were used for building a phylogenetic a tree with *Diaporthella corylina* as a root outgroup species [23]. The evolutionary relationships were taken on a five-gene concatenated alignment of ITS, *TEF1*, *CAL*, *HIS*, and *TUB* regions by maximum likelihood (ML) and maximum parsimony (MP) phylogenetic analyses. Sequences were aligned using the MAFFTv.7 online program with default parameters [27]. A partition homogeneity test implemented in PAUP* v.4.0 (Sinauer Associates, Sunderland, MA, USA) was applied to determine if the five sequence data could be combined. The best evolutionary model for the partitioning analysis was performed on the concatenated sequences by PartitionFinder 2.1.1 [28]. A concatenated alignment for the five gene regions was made from SequenceMatrix [29]. The inference methods of maximum likelihood and maximum parsimony in Mega 6.0 [30] were applied to estimate phylogeny for the concatenated sequences, with the evolutionary models GTR and AIC for ML and MP, respectively, with a bootstrap support of 1000 replicates. Evidence on the trees were visualized and edited by TreeGraph 2 [31].

### 2.4. Antifungal Activity Tests for Fungal VOCs

The antifungal activity of the VOCs was determined by the methods previously described [17,18,20]. The four endophytic fungal strains of *Diaporthe* and targeted plant pathogenic microorganisms were paired opposite to each other in Petri plates containing PDA with a diameter of 90 mm. The agar was divided into two halves by removing a 2 cm wide strip in the center. An endophytic test fungus was inoculated onto one half-moon of the agar and incubated at 25 °C for five days for optimum production of volatile compounds before the antagonism bioassay. A test pathogen was inoculated onto the opposite half-moon part of the agar at the fifth day. The plates were then wrapped with parafilm and incubated at 25 °C in dark for 72 h. Growth of filamentous pathogenic fungi were quantitatively assessed after 24 h, 48 h, and 72 h based on multiple measurements of growth relative to controls, as described previously [17,18]. The colony diameter was measured in an average of four diameters on hours 24, 48, and 72 h, disregarding the initial inoculum size. Percentage of growth inhibition was calculated as the formula: |(a − b/b)| × 100, a = mycelial colony diameter in control plate; b = mycelial colony diameter in the antagonism treatment plate. Statistical significance (*p* < 0.01) was evaluated by analysis of variance (ANOVA) followed by the Tukey 5% test. Antifungal activity of VOCs was tested against the plant pathogenic fungi *Alternaria alternata*, *Botryosphaeria dothidea*, *Botrytis cinerea*, *Cercospora* sp., *Colletotrichum gloeosporioides*, *Fusarium graminearum*, *Sphaeropsis sapinea*, and *Valsa sordida*, in addition to the oomycete *Phytophthora cinnamomi*. All tests were made in quintuplicate. Control cultures were obtained by growing each plant pathogen alone, under the same conditions.

### 2.5. Qualitative Analyses on Volatiles of the Four Endophytic Cultures

VOCs in the air space above the endophytic fungal colonies grown for five days at 25 ± 2 °C on PDA were analyzed using the solid phase microextraction (SPME) fiber technique according to previously described protocols [17,18,20]. Control PDA Petri plates not inoculated with the strain was used to subtract compounds contributed by the medium. All treatments and checks were done in triplicate. A fiber syringe of 50/30 divinylbenzene/carboxen on polydimethylsiloxane (Supelco, Bellefonte, PA, USA) was conditioned for 40 min at 200 °C, exposed to the vapor phase inside Petri during 40 min through a small hole (0.5 mm in diameter) drilled on the sides of the Petri plate. The fiber was directly inserted into the TRACE DSQ inlet (Thermo Electron Corporation, Beverly, MA, USA), at 200 °C, splitless mode. The desorption time was 40 s and the desorbed compounds were separated on a 30.0 m × 0.25 mm × 0.25 µm, HP-5MS capillary column, using the following GC oven temperature program: 2 min at 35 °C up to 220 °C at 7 °C/min. Helium was used as the carrier gas at a flow rate of 1 mL/min. The electronic ionization energy was 70 eV and the mass range scanned was 41–560 uma. The scan rate was 5 spec/s. Transfer line and ionization chamber temperatures were 250 °C and 200 °C respectively. Tentative identification of the volatile compounds produced by the four endophytic *Diaporthe* fungi was made via library comparison using the NIST database and all chemical compounds were described in this report following the NIST database chemical identity. Tentative compound identity was based on at least a 70% quality match with the NIST database information for each compound. Data acquisition and data processing were performed with the Hewlett Packard ChemStation software system (Version 2.0, Scientific Instrument Services, Inc., Ringoes, NJ, USA). Relative amounts of individual components of the treatments were determined and expressed as percentages of the peak area within the total peak area and as an average of the three replicates.

## 3. Results

### 3.1. The Identification on the Four Endophytic Isolates within the Diaporthe Genus

**Figure 1:**
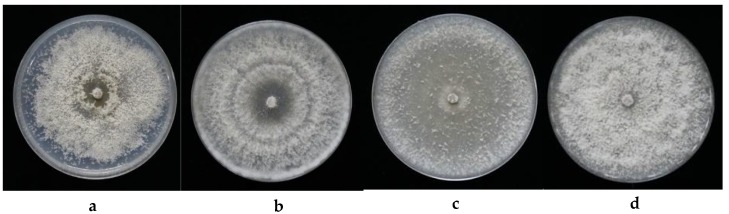
The colony cultures for the four endophytic *Diaporthe* fungi and their plant host. (**a**) FPYF3053; (**b**) FPYF3054; (**c**) FPYF3055; and (**d**) FPYF3056.

Each of the four isolates falling within the genus *Diaporthe* were further defined using molecular analyses as they appeared different, morphologically (Figure 1). For instance, strain FPYF3053 had compact mycelia with crenate margins, these colonies developed a brownish yellow pigmentation in the center on the underside having a growth rate of 18.3 mm day^−1^ (Figure 1a). On the other hand, strain FPYF3054 had aerial mycelium forming concentric rings with grey and dark pigmentation at the center showing a growth rate of 30.97 mm day^−1^ (Figure 1b). Strain FPYF3055 had vigorously-growing aerial hyphae near the margin, but loose hyphae scattered inside with aging, with a growth rate of 23 mm day^−1^ (Figure 1c). Finally, strain FPYF3056 had a compact mycelium with a crenate margin, but no pigmentation with a growth rate of 21.7 mm day^−1^ (Figure 1d).

**Figure 2:**
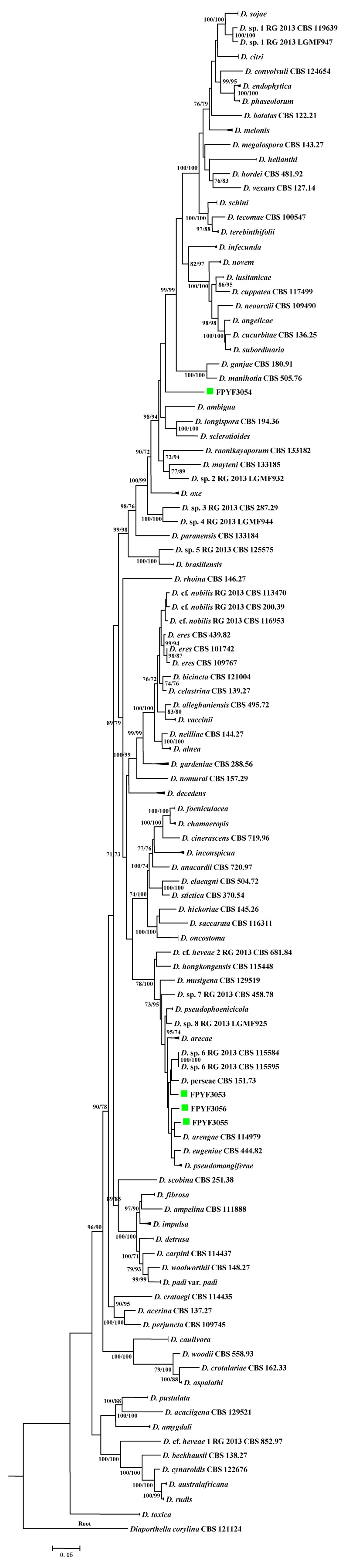
Phylogenetic tree based on combinedITS, *CAL*, *TEF1*, *HIS*, and *TUB* sequence alignment generated from a maximum parsimony and maximum likelihood analyses. Values near the branches represent parsimony/likelihood bootstrap support values (>70%), respectively. The tree is rooted with *Diaporthella corylina*. The four endophytic isolates were each named with strain ID marked green box. Compressed branches were used for saving space. The complete phylogenetic trees of MP and ML can be found in Figures S1 and S2, respectively.

A combined alignment of five loci ITS, *TUB*, *TEF1*, *HIS*, and *CAL* was used for ML and MP phylogenic analyses. Based on the multi-locus phylogeny (Figure 2), the four endophytic *Diaporthe* strains could not be placed in one species only because they are distinct from each other and from all reference species listed (Table 2, Figure 2). Strains FPYF3055 and FPYF3056 were clustered by giving a high bootstrap support (BS = 82) from MP inference (Figure S1) while both separated from each other in ML inference (Figure S2). The reference sequences used to construct the phylogenetic tree were listed in Table 2 with their Genbank accession numbers. The alignment was uploaded in Treebase assigned with SI 22757.

### 3.2. The VOCs’ Bioactivities of the Four Diaporthe Strains against Plant Fungal Pathogens

All of the four strains were observed to inhibit the growth of nine selected fungal pathogens by producing volatile compounds in the PDA medium (Table 3). The nine pathogens, *Alternaria alternata*, *Botryosphaeria dothidea*, *Botrytis cinerea*, *Cercospora asparagi*, *Colletotrichum gloeosporioides*, *Fusarium graminearum*, *Phytophthora cinnamomi*, *Sphaeropsis sapinea*, and *Valsa sordida*, are important causal agents to major trees, such as poplars and pines, or agricultural crops in China and elsewhere. All FPYF strains showed different inhibitory activities along the measurements, an exception was observed for the case of strain FPYF3053, which promoted the growth of *Phytophthora cinnamomi* (Table 3). Furthermore, all selected pathogens, except *V. sordid*, achieved obvious growth inhibition over around 10% during the testing period. After 24 h, *B. cinerea* was the most sensitive to VOCs emitted by all endophytic strains, reaching percent inhibitions of more than 55% when dual cultured with each strain. *B. dothidea* and *A. alternata* were highly sensitive to VOCs of all the endophytic strains, getting percent inhibitions of more than 30% with an exception to 28% of *A. alternata* in VOCs of the strain FPYF3053. *V. sordida* had the least sensitive or insensitive performance in VOCs from all the strains, showing percent inhibitions around 3% when dual cultured with FPYF3056. The inhibitive intensity of FPYF strains’ VOCs on growth of pathogens decreased in times to most duel cultures. The maximum drop of the intensity was by 31% in percent inhibition on the pathogen *B. cinerea* duel culturing with strain FPYF3056. The obvious increase in intensity occurred in the pathogen *F. graminearum* duel culturing with FPYF3055 and FPYF3056, increasing by around 10% during 72 h. Some pathogens grew fast without percent inhibition records after 24 h (*V. sordida*) or 72 h (*B. dothidea* and *F. graminearum*).

### 3.3. The Qualification on VOCs of the Four Endophytic Diaporthe Strains

Each of the *Diaporthe* isolates showed a unique VOC profile as measured by SPME (Table 4). Nineteen VOC components from the four fungi were identified and seven compounds were unidentified according our set standard of a 70% quality match with the GC-MS. Generally, the terpenoids were the major components in the VOCs of each strain. The main terpenes included α-thujene, β-phellandrene, γ-terpinene, l-menthone, cyclohexanol, 5-methyl-2-(1-methylethyl)-, α-muurolene. The amounts of each component of these monoterpenes had a relative area over 10% of the total of its VOCs. There also existed other minor terpenoids at very low amounts, including carene, α-phellandrene, thujone, caryophyllene, patchoulene, etc. Two monoterpenes, β-phellandrene and α-muurolene, and a chemical biphenylene,1,2,3,6,7,8,8a,8b-octahydro-4,5-dimethyl, which were detected in VOCs of all four strains. Four chemicals were common to VOCs from FPYF3053, FPYF3055, and FPYF3056, including α-thujene, 1,3-cyclohexadiene, 1-methyl-4-(1-methylethyl)-, γ-terpinene and 3-cyclohexen-1-ol, and 4-methyl-1-(1-methylethyl)-,(*R*)-. However, each strain produced a unique mixture of volatile organic compounds. The strain FPYF3053 produced 15 volatile compounds with three prominent components, α-thujene, β-phellandrene, and α-muurolene. FPYF3054 was able to synthesize eight compounds with three prominent components of β-phellandrene, l-menthone, and cyclohexanol,5-methyl-2-(1-methylethyl)- in VOC mixtures. Strains FPYF3055 and 3056 generated relatively close chemical compositions in amount and quality of VOCs compared to FPYF3053 and FPYF3054. However, FPYF3055 had three prominent components, α-thujene, γ-terpinene, and 3-cyclohexen-1-ol,4-methyl-1-(1-methylethyl)-,(*R*)-, in 12 compounds of the VOCs, while FPYF3056 had three prominent components—namely α-thujene β-phellandrene, and γ-terpinene—of 13 compounds in its VOCs.

## 4. Discussion

### 4.1. Endophytic Diaporthe spp. from Catharanthus roseus

Four isolates of endophytes in the genus *Diaporthe* were obtained from the medicinal plant *Catharanthus roseus* growing in a conservation area of Southern China. In order to best distinguish these individual organisms they were subjected to a combined analysis of five-loci alignment of *TEF1*-*TUB*-*CAL*-*HIS*-ITS which gave a more robust isolate identification [23]. Adding our four endophytic isolates did not affect the congruency in each locus, partition homogeneity for the combination and the best evolutionary model for the five-locus concatenated alignment reported. *Diaporthe* fungi are one of the most common endophytic fungal communites found in plants [11]. However, the previous work on endophytic fungi from *C. roseus* [7,8,32,33,34,35,36,37,38,39,40,41,42] did not record strains of the *Diaporthe* genus. *Alternaria alternata* was determined as the dominant endophytic species in leaf tissue of *C. roseus* along with associated fungi from the following genera, *Aspergillus*, *Fusarium*, *Penicillium*, and *Helminthosporium* [33]. In addition the endophytes of root tissue appeared including *Colletotrichum* sp., *Macrophomina phaseolina*, *Nigrospora sphaerica*, and *Fusarium solani* [7]. Other isolated endophytic fungi from this plant included *Colletotrichum truncatum*, *Drechsclera* sp., *Cladosporium* sp., and *Myrothecium* sp. [43]. To our four *Diaporthe* strains, no reproductive structures were obtained in the employed conditions. They were designated *Diaporthe* sp. strains (FPYF3053-3056) without spore characterization strictly using phylogenetic analysis. The strains seemed not to share a close phylogenetic relationship to any other species based on the five-locus alignment study (Figure 2, [12,23,26]). The robust inference on the strains will take place when fruits bodies appear combined with full species phylogeny in the genus *Diaporthe.*

### 4.2. VOCs Antifungal Effects of Endophytic Diaporthe spp. from Catharanthus roseus

Compounds extracted from *Catharanthus roseus* [4,5] and extracts from some endophytes of this plant [10,44] have been shown to have antimicrobial bioactivities to some human microbial pathogens and plant fungal pathogens, including *Staphylococcus aureus*, *Pseudomonas aeruginosa*, *Bacillus subtilis*, *Escherichia coli*, *Aspergillus fumigatus*, *Candida albicans*, etc. However, the VOCs or essential oils from *Catharanthus roseus* in the literature is scarce results on antimicrobial activities [45,46]. The previous work on the other endophytic fungi of this host plant did not consider that VOCs of the endophyte may have antimicrobial activities [7,8,32,33,34,35,36,37,38,39,40,41,42]. However, this work shows that VOCs produced by four endophytic *Diaporthe* fungi from the plant are able to functionally inhibit the growth of a number of specifically-targeted fungal pathogens (Table 3).

In the past there have been three endophytic *Diaporthe* strains recorded with their VOCs [18,19,47]. Two of them were reported to be inhibitory to plant pathogens [18,19]. One strain PR4 was isolated from a medicinal plant growing in Kashimir, Himalayas [19]; the other strain EC-4 was isolated from *Odontoglossum* sp. in Northern Ecuador [18]. With our four strains, the volatile compounds from endophytic *Diaporthe* fungi varied in degrees of inhibition against selected pathogenic fungi and test timings depending on the endophytic strain (Table 3 and Table 5). However, the maximal inhibition of fungal growth of *Diaporthe* was from strain PR4, which reduced growth of *Rhizoctonia solani* by 100%. FPYF strains’ and EC-4 VOCs also appeared effective in the inhibition of growth of *Botrytis cinerea* by more than 30% with a maximal of 50.42 ± 1.8%. During the test course of 72 h, to most cases, FPYF strains’ VOCs showed strong bioactivities in the first day and then decreased inhibition on the pathogens in following two days (Table 3). PR4 VOCs were effective in reducing radial growth of *Pythium ultimatum* by 13.3%; EC-4 VOCs were effective in reducing radial growth of *Pythium ultimatum*, *Phytophthora cinnamomi*, and *Phytophthora palmivora* by 59.1 ± 0.9%, 42.0 ± 0.5%, and 5.6 ± 0.5%, respectively. FPYF3054-3056’s VOCs were effective against *Phytophthora cinnamomi* in a range of 25.21 ± 4.3 ~ 11.32 ± 4.2%. The alcohol compounds such as 1-propanol,2-methyl- and 1-butanol,3-methyl- might made the oomycete *P. cinnamomi* more sensitive to EC-4’s VOCs [18], which were lack in VOCs of all FPYF strains (Table 4). The two alcohol compounds had antimicrobial activities in VOCs of endophytic *Phomopsis* sp. strain EC-4 [18]. The sensitivity of the pathogen *F. graminearum* to VOCs from *Diaprothe* spp. might be analogous even though the VOCs components were not similar among *Diaprothe* strains. Two *Diaporthe* strains FPYF3053, 3055 (Table 2) and *Diaporthe* strain PR4 [19] had percent inhibition of *F. graminearum* growth of around 30% under their VOCs bioactivities. However, only beta-phellandrene was a common compound found in VOCs among them (Table 4, [19]). Contrast to cytochalasins as a predominantly common component in soluble secondary metabolites of *Diaporthe* strains [16], the genus-specific or predominant conserved components of fungal VOCs of genus *Diaporthe* should be proposed to illustrate further. The experimental data suggests that the VOCs of FPYF strains are both biologically active and biologically selective. Finally, isolate FPYF3053 were showed no effective inhibition of *Phytophthora cinnamomi* growth. In this study, we attempt to understand the VOCs inhibitory impacts from the endophytic *Diaporthe* strains without consideration of interaction between the strains and pathogenes. Future research is proposed to investigate the dual interaction in the VOCs’ levels and other molecules between fungal interactions [48].

The headspace analyses of the four *Diaporthe* strains in potato dextrose medium revealed that three monoterpenes—β-phellandrene, biphenylene,1,2,3,6,7,8,8a,8b-octahydro-4,5-dimethyl and α-muurolene—seemed to be characteristic compounds of endophytic *Diaporthe* strains endophytic to *Catharathus roseus*. However, among all monoterpenes mentioned above, only 1-menthone can be found in volatile compounds of *Catharathus roseus* flowers, the essential oil of which is high in limonene and other monoterpenes [45,46]. Menthol and β-phellandrene were also found in VOCs of *Diaporthe* strain PR4 with very low relative amounts of less than 1.0% [19]. No chemicals were shared in VOCs between our FPYF strains and *Phomopsis* strain EC-4 (Table 4, [18]). Therefore, the antifungal VOCs from the four endophytic *Diaporthe* Chinese strains possesses unique VOC compositions compared with known *Diaporthe* VOCs. Although many fungi were reported to produce many terpene compounds in their VOCs [49], our *Diaporthe* fungi maybe of some interest as a source of some other monoterpenes, which often only have been thought to originate from specific plants. For instance, essential oils from many plants containing more or less such monoterpenes as α-thujene, β-phellandrene [50,51,52], γ-terpinene [53,54], l-menthone [55,56], cyclohexanol, α-muurolene, thujone, and caryophyllene have some antifungal activities. For example, γ-terpinene, singly or in mixtures with sabinene in oil from coastal redwood leaves, has strong antifungal activity on some endophytic fungi [53]. Therefore, it could be rational to infer the terpenes in FPYF strains synergistically played a main role in their inhibition pathogenic fungi growths. In addition, the high content of monoterpenes in the *Diaporthe* VOCs does have potential for the biofuel industry [18,20,57].

## Figures and Tables

**Table 1:** Access numbers for ITS, translation elongation factor 1-alpha (*TEF1*), beta-tubulin (*TUB*), histone H3 (*HIS*), calmodulin (*CAL*) genes region sequences of the four endophytic *Diaporthe* fungi in the GenBank of NCBI.

Isolate	ITS	*TEF1*	*Tublin*	*CAL*	*HIS*
FPYF3053	MH203054.1	MH220826.1	MH220836.1	MH220831.1	MH220839.1
FPYF3054	MH203055.1	MH220827.1	MH220833.1	MH220832.1	MH220840.1
FPYF3055	MH203056.1	MH220828.1	MH220834.1	MH220829.1	MH220837.1
FPYF3056	MH203057.1	MH220825.1	MH220835.1	MH220830.1	MH220838.1

**Table 2:** Reference sequences of *Diaporthe* strains with NCBI access numbers for phylogenetic analysis.

Source	ITS	*TEF1*	*TUB*	*CAL*	*HIS*
*Diaporthe acaciigena*_CBS 129521	KC343005.1	KC343731.1	KC343973.1	KC343247.1	KC343489.1
*Diaporthe acerina*_CBS 137.27	KC343006.1	KC343732.1	KC343974.1	KC343248.1	KC343490.1
*Diaporthe alleghaniensis*_CBS 495.72	KC343007.1	KC343733.1	KC343975.1	KC343249.1	KC343491.1
*Diaporthe alnea*_CBS 146.46	KC343008.1	KC343734.1	KC343976.1	KC343250.1	KC343492.1
*Diaporthe alnea*_CBS 159.47	KC343009.1	KC343735.1	KC343977.1	KC343251.1	KC343493.1
*Diaporthe ambigua*_CBS 114015	KC343010.1	KC343736.1	KC343978.1	KC343252.1	KC343494.1
*Diaporthe ambigua*_CBS 117167	KC343011.1	KC343737.1	KC343979.1	KC343253.1	KC343495.1
*Diaporthe amygdali*_CBS 126679	KC343022.1	KC343742.1	KC343984.1	KC343258.1	KC343506.1
*Diaporthe ampelina*_CBS 111888	KC343016.1	KC343748.1	KC343990.1	KC343264.1	KC343500.1
*Diaporthe amygdali_*CBS 111811	KC343019.1	KC343745.1	KC343987.1	KC343261.1	KC343503.1
*Diaporthe anacardii_*CBS 720.97	KC343024.1	KC343750.1	KC343992.1	KC343266.1	KC343508.1
*Diaporthe angelicae_*CBS 111592	KC343027.1	KC343753.1	KC343995.1	KC343269.1	KC343511.1
*Diaporthe angelicae_*CBS 123215	KC343028.1	KC343754.1	KC343996.1	KC343270.1	KC343512.1
*Diaporthe cucurbitae_*CBS 136.25	KC343031.1	KC343757.1	KC343999.1	KC343273.1	KC343515.1
*Diaporthe arecae_*CBS 161.64	KC343032.1	KC343758.1	KC344000.1	KC343274.1	KC343516.1
*Diaporthe arecae*_CBS 535.75	KC343033.1	KC343759.1	KC344001.1	KC343275.1	KC343517.1
*Diaporthe arengae*_CBS 114979	KC343034.1	KC343760.1	KC344002.1	KC343276.1	KC343518.1
*Diaporthe aspalathi_*CBS 117169	KC343036.1	KC343762.1	KC344004.1	KC343278.1	KC343520.1
*Diaporthe aspalathi_*CBS 117168	KC343035.1	KC343761.1	KC344003.1	KC343277.1	KC343519.1
*Diaporthe australafricana_*CBS 111886	KC343038.1	KC343764.1	KC344006.1	KC343280.1	KC343522.1
*Diaporthe australafricana_*CBS 113487	KC343039.1	KC343765.1	KC344007.1	KC343281.1	KC343523.1
*Diaporthe batatas_*CBS 122.21	KC343040.1	KC343766.1	KC344008.1	KC343282.1	KC343524.1
*Diaporthe beckhausii_*CBS 138.27	KC343041.1	KC343767.1	KC344009.1	KC343283.1	KC343525.1
*Diaporthe bicincta*_CBS 121004	KC343134.1	KC343860.1	KC344102.1	KC343376.1	KC343618.1
*Diaporthe brasiliensis*_CBS 133183	KC343042.1	KC343768.1	KC344010.1	KC343284.1	KC343526.1
*Diaporthe brasiliensis*_LGMF926	KC343043.1	KC343769.1	KC344011.1	KC343285.1	KC343527.1
*Diaporthe carpini*_CBS 114437	KC343044.1	KC343770.1	KC344012.1	KC343286.1	KC343528.1
*Diaporthe caulivora*_CBS 127268	KC343045.1	KC343771.1	KC344013.1	KC343287.1	KC343529.1
*Diaporthe caulivora*_CBS 178.55	KC343046.1	KC343772.1	KC344014.1	KC343288.1	KC343530.1
*Diaporthe celastrina*_CBS 139.27	KC343047.1	KC343773.1	KC344015.1	KC343289.1	KC343531.1
*Diaporthe chamaeropis*_CBS 454.81	KC343048.1	KC343774.1	KC344016.1	KC343290.1	KC343532.1
*Diaporthe chamaeropis*_CBS 753.70	KC343049.1	KC343775.1	KC344017.1	KC343291.1	KC343533.1
*Diaporthe cinerascens*_CBS 719.96	KC343050.1	KC343776.1	KC344018.1	KC343292.1	KC343534.1
*Diaporthe citri*_CBS 199.39	KC343051.1	KC343777.1	KC344019.1	KC343293.1	KC343535.1
*Diaporthe citri*_CBS 230.52	KC343052.1	KC343778.1	KC344020.1	KC343294.1	KC343536.1
*Diaporthe convolvuli*_CBS 124654	KC343054.1	KC343780.1	KC344022.1	KC343296.1	KC343538.1
*Diaporthe crataegi_*CBS 114435	KC343055.1	KC343781.1	KC344023.1	KC343297.1	KC343539.1
*Diaporthe crotalariae*_CBS 162.33	KC343056.1	KC343782.1	KC344024.1	KC343298.1	KC343540.1
*Diaporthe cuppatea_*CBS 117499	KC343057.1	KC343783.1	KC344025.1	KC343299.1	KC343541.1
*Diaporthe cynaroidis*_CBS 122676	KC343058.1	KC343784.1	KC344026.1	KC343300.1	KC343542.1
*Diaporthe decedens_*CBS 109772	KC343059.1	KC343785.1	KC344027.1	KC343301.1	KC343543.1
*Diaporthe decedens*_CBS 114281	KC343060.1	KC343786.1	KC344028.1	KC343302.1	KC343544.1
*Diaporthe detrusa*_CBS 109770	KC343061.1	KC343787.1	KC344029.1	KC343303.1	KC343545.1
*Diaporthe detrusa_*CBS 114652	KC343062.1	KC343788.1	KC344030.1	KC343304.1	KC343546.1
*Diaporthe elaeagni_*CBS 504.72	KC343064.1	KC343790.1	KC344032.1	KC343306.1	KC343548.1
*Diaporthe endophytica*_CBS 133811	KC343065.1	KC343791.1	KC344033.1	KC343307.1	KC343549.1
*Diaporthe endophytica*_LGMF928	KC343068.1	KC343794.1	KC344036.1	KC343310.1	KC343552.1
*Diaporthe eres*_CBS 439.82	KC343090.1	KC343816.1	KC344058.1	KC343332.1	KC343574.1
*Diaporthe eres*_CBS 101742	KC343073.1	KC343799.1	KC344041.1	KC343315.1	KC343557.1
*Diaporthe eres*_CBS 109767	KC343075.1	KC343801.1	KC344043.1	KC343317.1	KC343559.1
*Diaporthe* cf. *nobilis* RG-2013_CBS 113470	KC343146.1	KC343872.1	KC344114.1	KC343388.1	KC343630.1
*Diaporthe* cf. *nobilis* RG-2013_CBS 116953	KC343147.1	KC343873.1	KC344115.1	KC343389.1	KC343631.1
*Diaporthe* cf. *nobilis* RG-2013_CBS 200.39	KC343151.1	KC343877.1	KC344119.1	KC343393.1	KC343635.1
*Diaporthe eugeniae*_CBS 444.82	KC343098.1	KC343824.1	KC344066.1	KC343340.1	KC343582.1
*Diaporthe fibrosa*_CBS 109751	KC343099.1	KC343825.1	KC344067.1	KC343341.1	KC343583.1
*Diaporthe fibrosa*_CBS 113830	KC343100.1	KC343826.1	KC344068.1	KC343342.1	KC343584.1
*Diaporthe foeniculacea*_CBS 123208	KC343104.1	KC343830.1	KC344072.1	KC343346.1	KC343588.1
*Diaporthe foeniculacea*_CBS 111553	KC343101.1	KC343827.1	KC344069.1	KC343349.1	KC343585.1
*Diaporthe foeniculacea*_CBS 187.27	KC343107.1	KC343833.1	KC344075.1	KC343343.1	KC343591.1
*Diaporthe ganjae*_CBS 180.91	KC343112.1	KC343838.1	KC344080.1	KC343354.1	KC343596.1
*Diaporthe gardeniae*_CBS 288.56	KC343113.1	KC343839.1	KC344081.1	KC343355.1	KC343597.1
*Diaporthe helianthi*_CBS 592.81	KC343115.1	KC343841.1	KC344083.1	KC343357.1	KC343599.1
*Diaporthe helianthi*_CBS 344.94	KC343114.1	KC343840.1	KC344082.1	KC343356.1	KC343598.1
*Diaporthe hickoriae*_CBS 145.26	KC343118.1	KC343844.1	KC344086.1	KC343360.1	KC343602.1
*Diaporthe hongkongensis*_CBS 115448	KC343119.1	KC343845.1	KC344087.1	KC343361.1	KC343603.1
*Diaporthe hordei*_CBS 481.92	KC343120.1	KC343846.1	KC344088.1	KC343362.1	KC343604.1
*Diaporthe impulsa_*CBS 114434	KC343121.1	KC343847.1	KC344089.1	KC343363.1	KC343605.1
*Diaporthe impulsa*_CBS 141.27	KC343122.1	KC343848.1	KC344090.1	KC343364.1	KC343606.1
*Diaporthe inconspicua*_LGMF922	KC343124.1	KC343849.1	KC344091.1	KC343365.1	KC343607.1
*Diaporthe inconspicua*_CBS 133813	KC343123.1	KC343850.1	KC344092.1	KC343366.1	KC343608.1
*Diaporthe infecunda*_CBS 133812	KC343126.1	KC343852.1	KC344094.1	KC343368.1	KC343610.1
*Diaporthe infecunda*_LGMF933	KC343132.1	KC343858.1	KC344100.1	KC343374.1	KC343616.1
*Diaporthe longispora*_CBS 194.36	KC343135.1	KC343861.1	KC344103.1	KC343377.1	KC343619.1
*Diaporthe lusitanicae*_CBS 123212	KC343136.1	KC343862.1	KC344104.1	KC343378.1	KC343620.1
*Diaporthe lusitanicae_*CBS 123213	KC343137.1	KC343863.1	KC344105.1	KC343379.1	KC343621.1
*Diaporthe manihotia*_CBS 505.76	KC343138.1	KC343864.1	KC344106.1	KC343380.1	KC343622.1
*Diaporthe mayteni*_CBS 133185	KC343139.1	KC343865.1	KC344107.1	KC343381.1	KC343623.1
*Diaporthe megalospora*_CBS 143.27	KC343140.1	KC343866.1	KC344108.1	KC343383.1	KC343624.1
*Diaporthe melonis*_CBS 507.78	KC343142.1	KC343868.1	KC344110.1	KC343384.1	KC343626.1
*Diaporthe melonis*_CBS 435.87	KC343141.1	KC343867.1	KC344109.1	KC343382.1	KC343625.1
*Diaporthe musigena*_CBS 129519	KC343143.1	KC343869.1	KC344111.1	KC343385.1	KC343627.1
*Diaporthe neilliae*_CBS 144.27	KC343144.1	KC343870.1	KC344112.1	KC343386.1	KC343628.1
*Diaporthe neoarctii*_CBS 109490	KC343145.1	KC343871.1	KC344113.1	KC343387.1	KC343629.1
*Diaporthe nomurai*_CBS 157.29	KC343154.1	KC343880.1	KC344122.1	KC343396.1	KC343638.1
*Diaporthe novem*_CBS 127270	KC343156.1	KC343882.1	KC344124.1	KC343398.1	KC343640.1
*Diaporthe novem*_CBS 354.71	KC343158.1	KC343884.1	KC344126.1	KC343400.1	KC343642.1
*Diaporthe oncostoma*_CBS 109741	KC343161.1	KC343887.1	KC344129.1	KC343403.1	KC343645.1
*Diaporthe oncostoma*_CBS 100454	KC343160.1	KC343886.1	KC344128.1	KC343402.1	KC343644.1
*Diaporthe oxe*_CBS 133186	KC343164.1	KC343890.1	KC344132.1	KC343406.1	KC343648.1
*Diaporthe oxe_*CBS 133187	KC343165.1	KC343891.1	KC344133.1	KC343407.1	KC343649.1
*Diaporthe padi* var. *padi_*CBS 114200	KC343169.1	KC343895.1	KC344137.1	KC343411.1	KC343653.1
*Diaporthe padi* var. *padi_*CBS 114649	KC343170.1	KC343896.1	KC344138.1	KC343412.1	KC343654.1
*Diaporthe paranensis_*CBS 133184	KC343171.1	KC343897.1	KC344139.1	KC343413.1	KC343655.1
*Diaporthe perjuncta_*CBS 109745	KC343172.1	KC343898.1	KC344140.1	KC343414.1	KC343656.1
*Diaporthe perseae*_CBS 151.73	KC343173.1	KC343899.1	KC344141.1	KC343415.1	KC343657.1
*Diaporthe phaseolorum*_CBS 116019	KC343175.1	KC343901.1	KC344143.1	KC343417.1	KC343659.1
*Diaporthe phaseolorum*_CBS 116020	KC343176.1	KC343902.1	KC344144.1	KC343418.1	KC343660.1
*Diaporthe pseudomangiferae*_CBS 101339	KC343181.1	KC343907.1	KC344149.1	KC343423.1	KC343665.1
*Diaporthe pseudomangiferae*_CBS 388.89	KC343182.1	KC343908.1	KC344150.1	KC343424.1	KC343666.1
*Diaporthe pseudophoenicicola*_CBS 462.69	KC343184.1	KC343910.1	KC344152.1	KC343426.1	KC343668.1
*Diaporthe pseudophoenicicola*_CBS 176.77	KC343183.1	KC343909.1	KC344151.1	KC343425.1	KC343667.1
*Diaporthe pustulata*_CBS 109784	KC343187.1	KC343913.1	KC344155.1	KC343429.1	KC343671.1
*Diaporthe pustulata*_CBS 109742	KC343185.1	KC343911.1	KC344153.1	KC343427.1	KC343669.1
*Diaporthe raonikayaporum*_CBS 133182	KC343188.1	KC343914.1	KC344156.1	KC343430.1	KC343672.1
*Diaporthe rhoina_*CBS 146.27	KC343189.1	KC343915.1	KC344157.1	KC343431.1	KC343673.1
*Diaporthe saccarata_*CBS 116311	KC343190.1	KC343916.1	KC344158.1	KC343432.1	KC343674.1
*Diaporthe schini_C*BS 133181	KC343191.1	KC343917.1	KC344159.1	KC343433.1	KC343675.1
*Diaporthe schini*_LGMF910	KC343192.1	KC343918.1	KC344160.1	KC343434.1	KC343676.1
*Diaporthe sclerotioides*_CBS 296.67	KC343193.1	KC343919.1	KC344161.1	KC343435.1	KC343677.1
*Diaporthe sclerotioides*_CBS 710.76	KC343194.1	KC343920.1	KC344162.1	KC343436.1	KC343678.1
*Diaporthe scobina*_CBS 251.38	KC343195.1	KC343921.1	KC344163.1	KC343437.1	KC343679.1
*Diaporthe sojae*_CBS 100.87	KC343196.1	KC343922.1	KC344164.1	KC343438.1	KC343680.1
*Diaporthe longicolla* isolate PL4	HM347700.1	HM347685.1	KC344167.1	KC343441.1	KC343683.1
*Diaporthe sojae*_CBS 116017	KC343197.1	KC343923.1	KC344165.1	KC343439.1	KC343681.1
*Diaporthe sojae*_CBS 180.55	KC343200.1	KC343926.1	KC344168.1	KC343442.1	KC343684.1
*Diaporthe subordinaria*_CBS 101711	KC343213.1	KC343938.1	KC344180.1	KC343454.1	KC343696.1
*Diaporthe subordinaria*_CBS 464.90	KC343214.1	KC343939.1	KC344181.1	KC343455.1	KC343697.1
*Diaporthe tecomae*_CBS 100547	KC343215.1	KC343940.1	KC344182.1	KC343456.1	KC343698.1
*Diaporthe terebinthifolii*_CBS 133180	KC343216.1	KC343941.1	KC344184.1	KC343457.1	KC343699.1
*Diaporthe terebinthifolii*_LGMF907	KC343217.1	KC343942.1	KC344183.1	KC343458.1	KC343700.1
*Diaporthe toxica*_CBS 534.93	KC343220.1	KC343943.1	KC344185.1	KC343459.1	KC343701.1
*Diaporthe toxica*_CBS 535.93	KC343221.1	KC343946.1	KC344188.1	KC343462.1	KC343704.1
*Diaporthe vaccinii_*CBS 160.32	KC343228.1	KC343947.1	KC344189.1	KC343463.1	KC343705.1
*Diaporthe vaccinii*_CBS 122112	KC343224.1	KC343954.1	KC344196.1	KC343470.1	KC343712.1
*Diaporthe vexans*_CBS 127.14	KC343229.1	KC343950.1	KC344192.1	KC343466.1	KC343708.1
*Diaporthe rudis*_CBS 113201	KC343234.1	KC343955.1	KC344197.1	KC343471.1	KC343713.1
*Diaporthe rudis*_CBS 109768	KC343233.1	KC343960.1	KC344202.1	KC343476.1	KC343718.1
*Diaporthe woodii_*CBS 558.93	KC343244.1	KC343959.1	KC344201.1	KC343475.1	KC343717.1
*Diaporthe woolworthii*_CBS 148.27	KC343245.1	KC343970.1	KC344212.1	KC343486.1	KC343728.1
*Diaporthe* cf. *heveae* 1 RG-2013_CBS 852.97	KC343116.1	KC343971.1	KC344213.1	KC343487.1	KC343729.1
*Diaporthe* cf. *heveae* 2 RG-2013_CBS 681.84	KC343117.1	KC343842.1	KC344084.1	KC343358.1	KC343600.1
*Diaporthe* sp. 1 RG-2013_CBS 119639	KC343202.1	KC343843.1	KC344085.1	KC343359.1	KC343601.1
*Diaporthe* sp. 1 RG-2013_LGMF947	KC343203.1	KC343928.1	KC344170.1	KC343444.1	KC343686.1
*Diaporthe* sp. 2 RG-2013_LGMF932	KC343204.1	KC343929.1	KC344171.1	KC343445.1	KC343687.1
*Diaporthe* sp. 3 RG-2013_CBS 287.29	KC343205.1	KC343930.1	KC344172.1	KC343446.1	KC343688.1
*Diaporthe* sp. 4 RG-2013_LGMF944	KC343206.1	KC343931.1	KC344173.1	KC343448.1	KC343689.1
*Diaporthe* sp. 5 RG-2013_CBS 125575	KC343207.1	KC343932.1	KC344174.1	KC343447.1	KC343690.1
*Diaporthe* sp. 6 RG-2013_CBS 115584	KC343208.1	KC343933.1	KC344175.1	KC343449.1	KC343691.1
*Diaporthe* sp. 6 RG-2013_CBS 115595	KC343209.1	KC343934.1	KC344176.1	KC343450.1	KC343692.1
*Diaporthe* sp. 7 RG-2013_CBS 458.78	KC343210.1	KC343935.1	KC344177.1	KC343451.1	KC343693.1
*Diaporthe* sp. 8 RG-2013_LGMF925	KC343211.1	KC343936.1	KC344178.1	KC343452.1	KC343694.1
*Diaporthella corylina*_CBS 121124	KC343004.1	KC343937.1	KC344179.1	KC343453.1	KC343695.1
*Diaporthe stictica_*CBS 370.54	KC343212.1	KC343730.1	KC343972.1	KC343246.1	KC343488.1

**Table 3:** Growth inhibition percentage of plant pathogens by VOC bioassays of four *Diaporthe* strains. Percent of inhibition is shown as the means of four measurements of diameters with standard deviation (*n* = 4).

Pathogen	Day	FPYF3053		FPYF3054		FPYF3055		FPYF3056	
		Percentage Inhibition	*p*-Value	Percentage Inhibition	*p*-Value	Percentage Inhibition	*p*-Value	Percentage Inhibition	*p*-Value
*Alternaria alternata*	24 h	28.77 ± 2.26	0.0003	41.41 ± 1.65	0.0001	37.68 ± 5.6	0.0078	34.51 ± 2.03	0.0002
	48 h	22.42 ± 2.34	0.0003	30.19 ± 1.56	0.0009	30.25 ± 5.12	0.0113	26.50 ± 3.42	0.0006
	72 h	15.25 ± 2.59	0.0019	23.43 ± 2.27	0.0039	22 ± 4.03	0.0148	16.34 ± 2.36	0.0010
*Botryosphaeria dothidea*	24 h	46.3 ± 4.30	0.0030	50.17 ± 2.43	0.0006	43.74 ± 2.15	0.0000	37.88 ± 3.80	0.0002
	48 h	45.28 ± 2.63	0.0000	45.14 ± 2.35	0.0000	42.78 ± 0.43	0.0000	38.99 ± 0.98	0.0000
	72 h	NE *							
*Botrytis cinerea*	24 h	64.47 ± 1.05	0.0000	55.26 ± 4.82	0.0000	60.72 ± 1.91	0.0001	55.26 ± 4.71	0.0000
	48 h	50.42 ± 1.79	0.0000	35.02 ± 1.22	0.0000	39.44 ± 3.70	0.0000	32.41 ± 3.75	0.0003
	72 h	36.55 ± 2.81	0.0000	24.27 ± 3.08	0.0001	30.10 ± 3.54	0.0005	24.25 ± 4.62	0.0025
*Cercospora asparagi*	24 h	31.46 ± 4.11	0.0003	22.64 ± 2.86	0.0074	23.21 ± 4.54	0.0202	18.57 ± 5.01	0.0086
	48 h	33.81 ± 2.97	0.0000	24.32 ± 2.21	0.0008	22.02 ± 2.96	0.0000	16.34 ± 1.53	0.0000
	72 h	23.32 ± 2.17	0.0007	19.09 ± 2.73	0.0031	11.94 ± 3.54	0.0083	4.56 ± 0.85	0.0032
*Colletotrichum gloeosporioides*	24 h	26.83 ± 4.78	0.0153	9.75 ± 2.33	0.0009	10.68 ± 1.14	0.0001	10.74 ± 2.38	0.0229
	48 h	20.94 ± 3.33	0.0051	11.76 ± 2.35	0.0006	8.91 ± 1.24	0.0001	9.78 ± 2.36	0.0017
	72 h	20.68 ± 1.56	0.0024	9.39 ± 2.78	0.0023	6.29 ± 0.80	0.0001	6.82 ± 2.01	0.0388
*Fusarium graminearum*	24 h	25.68 ± 1.13	0.0031	14.68 ± 2.05	0.0147	20.45 ± 3.62	0.0046	12.96 ± 1.42	0.0103
	48 h	29.99 ± 5.29	0.0086	12.9 ± 4.50	0.0284	31.12 ± 3.57	0.0203	21.59 ± 4.57	0.0425
	72 h	NE							
*Phytophthora cinnamomi*	24 h	−5.01 ± 1.14 **	0.0029	19.21 ± 4.54	0.0036	31.02 ± 2.58	0.0001	8.38 ± 3.10	0.0154
	48 h	−15.65 ± 6.36	0.0186	12.19 ± 3.30	0.0500	25.21 ± 4.29	0.0050	11.32 ± 4.22	0.0302
	72 h	−19.70 ± 4.19	0.0153	11.91 ± 2.12	0.0209	21.03 ± 2.80	0.0031	8.94 ± 2.03	0.0013
*Sphaeropsis sapinea*	24 h	23.39 ± 4.25	0.0147	22.69 ± 5.23	0.0239	21.84 ± 7.61	0.0491	7.41 ± 2.68	0.0364
	48 h	20.93 ± 1.04	0.0009	23.85 ± 1.68	0.0023	18.33 ± 5.22	0.0367	9.53 ± 0.60	0.0024
	72 h	NE							
*Valsa sordida*	24 h	5.96 ± 1.61	0.0115	9.73 ± 2.79	0.0014	5.14 ± 1.02	0.0153	3.15 ± 1.00	0.0177
	48 h	NE							
	72 h	NE							

* No data. ** Negative values mean growth stimulation.

**Table 4:** Chemical composition of volatiles obtained from mycelial cultures of the four endophytic *Diaporthe* fungi using solid–phase microextraction (SPME).

Retention Time (min)	Molecular Weight	Compound	Quality (%) ^a^				Abundance (Relative) ^b^			
			FPYF3053	FPYF3054	FPYF3055	FPYF3056	FPYF3053	FPYF3054	FPYF3055	FPYF3056
6.17	106	Ethylbenzene		91.8	75.5	77.9		0.42	0.80	0.92
7.67	136	α-Thujene	91.9		89.3	89.8	30.57		37.10	36.19
9.88	136	1,3-Cyclohexadiene, 1-methyl-4-(1-methylethyl)-	84.7		80.2	81.2	6.26		5.92	1.87
9.89	136	2-Carene	86			84.9	1.80			6.20
10.21	136	α-Phellandrene		74.9	78			0.74	5.15	
10.22	136	β-Phellandrene	88.4	90.9	75.2	87.7	12.55	56.07	2.35	18.70
10.92	136	γ-Terpinene	89.4		85.6	88.7	21.15		16.82	19.21
11.63	136	Cyclohexene, 1-methyl-4-(1-methylethylidene)-			81.1				1.66	
11.89	154	2-Cyclohexen-1-ol, 1-methyl-4-(1-methylethyl)-	81				0.65			
12.29	152	Unknown	63.4		68.2		0.29		1.24	
12.3	152	Thujone				71.5				0.65
13.21	154	l-Menthone		90.4				27.91		
13.68	156	Cyclohexanol, 5-methyl-2-(1-methylethyl)-		87.6				10.29		
13.76	154	3-Cyclohexen-1-ol, 4-methyl-1-(1-methylethyl)-, (*R*)-	88.5		89.7	85.6	5.53		22.38	5.88
13.91	208	2,4,4-Trimethyl-3-(3-methylbutyl)cyclohex-2-enone		70.1				0.44		
14.12	352	Unknown	66.3				0.28			
14.13	240	Unknown				64.4				0.59
14.14	170	Unknown			64.9				1.35	
17.42	388	Unknown				60.9				0.31
18.41	188	Biphenylene, 1,2,3,6,7,8,8a,8b-octahydro-4,5-dimethyl-	79	73.9	68.5	72.2	7.05	1.62	2.06	6.81
18.92	204	Caryophyllene	73.6				0.46			
19.11	204	Unknown	69.7				0.31			
19.74	204	Patchoulene	76.8				0.45			
20.01	222	Unknown				63.1				0.41
20.13	204	Cedrene	78.2				1.11			
20.46	204	α-Muurolene	92.2	81.1	81	81.6	11.54	2.51	3.17	2.26

Notes: Data are averages of two cultures grown on the same medium with subtracting those from the control PDA plate. ^a^ The quality match is the % likelihood that the compound is identical to that which is listed on the table based on the NIST database. Compounds assigned as unknown with lower than 70% quality match. ^b^ The abundance figure presents the percentage amount of each compound in total area relative to all listed compounds detected for one strain.

**Table 5:** Comparison VOCs’ inhibitive effect among *Diaporthe* strains.

Pathogens	Percent Growth Inhibition		
	*Phomopsis* sp. EC-4 [17] *	*Diaporthe* Strain PR4 [18]	FPYF3053-3056 **
*Aspergillus flavus*	/ ***	34.6	/
*Aspergillus fumigatus*	57.0 ± 0.5	/	/
*Alternaria alternata*	/	/	30.25 ± 5.1~22.42 ± 2.3
*Botryosphaeria dothidea*	/	/	45.14 ± 2.4~25.28 ± 2.6
*Botrytis cinerea*	37.8 ± 0.5	/	50.42 ± 1.8~32.41 ± 3.8
*Ceratocystis fimbriata*	/	0.0	/
*Ceratocystis ulmi*	11.1 ± 1.5	/	/
*Cercospora asparagi*	/	/	33.81 ± 2.97~16.34 ± 1.5
*Cercospora beticola*	19.5 ± 0.5	/	/
*Colletotrichum* sp.	/	/	20.94 ± 3.3~8.91 ± 2.4
*Colletotrichum lagenarium*	0.0	/	/
*Fusarium oxysporum*	/	34.6	31.12 ± 3.6~12.9 ± 4.5
*Fusarium solani*	43.2 ± 0.00	16.6	/
*Geotrichum candidum,*	45.3 ± 0.5	57.0	/
*Trichoderma viride*	0.0	/	/
*Rhizoctonia solani*	53.0 ± 1.0	100	/
*Sphaerospsis sapinea*	/	/	23.85 ± 1.7~9.53 ± 0.6
*Sclerotinia sclerotiorum*	70.7 ± 1.1	/	/
*Valsa sordida*	/	/	9.73 ± 2.8~3.15 ± 1.00
*Verticillium dahliae*	19.4 ± 0.0	0.0	/
*Pythium ultimatum*	59.1 ± 0.9	13.3	/
*Phytophthora cinnamomi*	42.0 ± 0.5	/	−19.70 ± 4.19~−5.01 ± 1.14, 25.21 ± 4.3~11.32 ± 4.2
*Phytophthora palmivora*	5.6 ± 0.5	/	/

* Data reference, ** the values listed as range for the four strains during 72 h, *** no data.

## Supplementary Materials

The following are available online at http://www.mdpi.com/2309-608X/4/2/65/s1. Figure S1. MP phylogenetic tree of five-locus alignment for FPYF3053-3056 in genus *Diaporthe* with *Diaporthella corylina* as an outgroup; Figure S2. ML phylogenetic tree of five-locus alignment for FPYF3053-3056 in genus *Diaporthe* with *Diaporthella corylina* as an outgroup.
