# Supplementary figures and images for: Antifungal Activities of Volatile Secondary Metabolites of Four Diaporthe Strains Isolated from Catharanthus roseus
Source: J Fungi (Basel). 2018 May 30;4(2):65. doi: 10.3390/jof4020065 (PMC6023506; doi:10.3390/jof4020065)

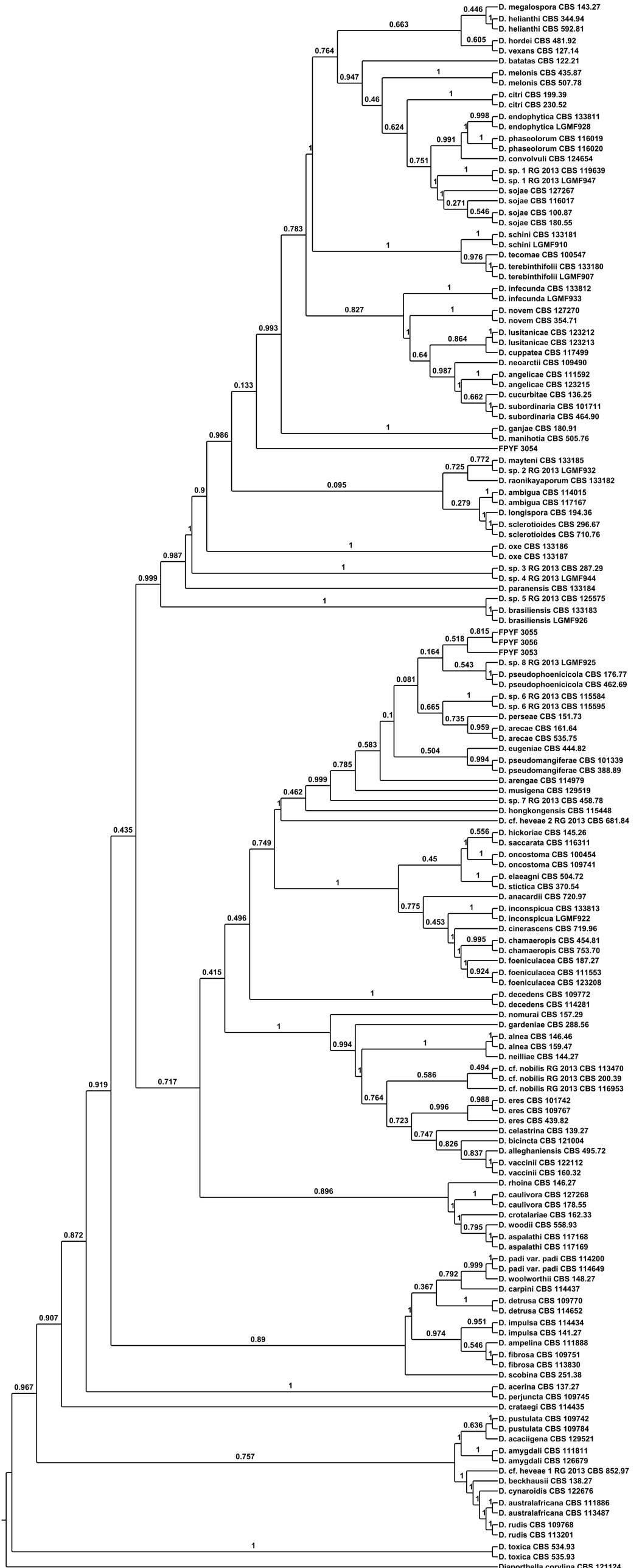

Supplement: Supplementary file 1 [file jof-04-00065-s001.zip › Supplementary Figure 1.pdf]

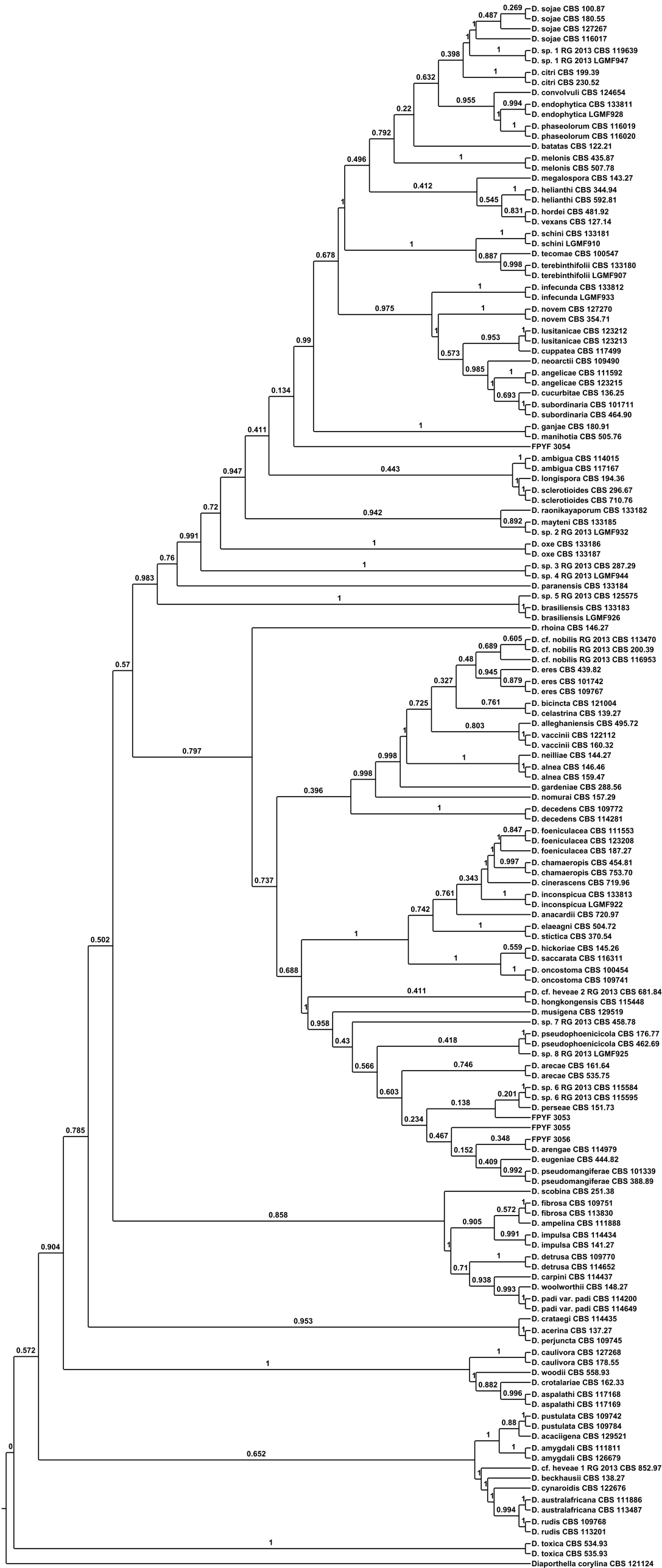

Supplement: Supplementary file 1 [file jof-04-00065-s001.zip › Supplementary Figure 2.pdf]
